# Long-term impact of a faculty mentoring program in academic medicine

**DOI:** 10.1371/journal.pone.0207634

**Published:** 2018-11-29

**Authors:** Jason A. Efstathiou, Michael R. Drumm, Jonathan P. Paly, Donna M. Lawton, Regina M. O’Neill, Andrzej Niemierko, Lisa R. Leffert, Jay S. Loeffler, Helen A. Shih

**Affiliations:** 1 Department of Radiation Oncology, Massachusetts General Hospital, Harvard Medical School, Boston, Massachusetts, United States of America; 2 Center for Faculty Development, Massachusetts General Hospital, Boston, Massachusetts, United States of America; 3 Sawyer Business School, Suffolk University, Boston, Massachusetts, United States of America; 4 Department of Radiation Oncology, Division of Biostatistics, Massachusetts General Hospital, Boston, Massachusetts, United States of America; 5 Department of Anesthesia, Critical Care & Pain Medicine, Massachusetts General Hospital, Harvard Medical School, Boston, Massachusetts, United States of America; Indiana University, UNITED STATES

## Abstract

The authors conducted a prospective longitudinal study from 2009 to 2016 to assess the short and long-term impact of a formal mentorship program on junior faculty satisfaction and productivity. Junior faculty mentees enrolled in the program and junior faculty without formal mentorship were administered surveys before and after the program to assess satisfaction with their mentoring experiences. Long-term retention, promotion, and funding data were also collected. Twenty-three junior faculty mentees and 91 junior faculty controls were included in the study. Mentees came from the Departments of Radiation Oncology and Anesthesia, Critical Care, and Pain Management. After participating in the mentorship program, mentees demonstrated an increase in satisfaction from baseline in five of seven domains related to mentoring, while controls experienced no significant change in satisfaction in six of the seven domains. At long-term follow up, mentees were more likely than controls to hold senior faculty positions (percent senior faculty: 47% vs. 13%, p = 0.030) despite no difference in initial administrative rank. When comparing the subset of faculty who were Instructors at baseline, mentees were more likely to be funded and/or promoted than controls (p = 0.030). A majority of mentees reported that the program strengthened their long-term success, and many maintained their original mentoring relationships and formed new ones, highlighting the strong culture of mentorship that was instilled. Several short-term and long-term benefits were fostered from this formal mentorship program. These findings highlight the potential impact of mentorship programs in propagating a culture of mentorship and excellence.

## Introduction

Mentorship often assumes an integral role in the mission of academic medical centers because it is considered synonymous with the growth and development of junior faculty. Not simply counseling or teaching, mentoring is a complex interaction grounded by a close relationship between a junior mentee and a senior mentor [1]. Many reports have sought to establish the required attributes of a successful mentoring relationship, which may encompass respect, proximity, comprehensive feedback, and mentor experience [1–4]. Specifically, effective mentors in academic medicine demonstrate expertise, honesty, accessibility, approachability, and supportiveness. These characteristics enable mentors to provide guidance, resources, encouragement, constructive criticism, and support to their mentees [5,6]. A successful mentoring relationship is not solely contingent on what mentors can offer their mentees; mentees must be proactive, receptive to feedback, self-reflective, and committed to sustaining the mentoring relationship [4,6,7]. This relationship can either be informally established by faculty themselves or formally organized by an academic medical center [8,9]. Given their potential benefits, faculty and organizations are increasingly interested in establishing mentoring programs [10].

A successful mentoring relationship is well poised to fulfill several needs of junior faculty. Mentees often express interest in receiving career development support from mentors, including building a professional network, publishing research, and applying for grants [6]. Some studies have reported tangible benefits of mentorship on junior faculty development, including improved satisfaction, publication quality, and time to promotion [11–13]. However, a systematic review of the literature on mentoring in academic medicine showed that there is a wide variability in how reports define a mentoring relationship and that the reports were mostly cross-sectional self-report surveys, limiting the ability to draw firm conclusions regarding the benefit of mentoring in academic institutions [2].

Considering the many competing demands of mentors and medical centers, precision about the short-term and long-term effects of a formal mentoring program is especially important to guide the allocation of resources to these programs and ensure that cost is proportional to benefit [13–14]. Specifically, there is a need for research on the benefits of mentoring in academic medical centers that spans a multi-year time frame and is informed by both objective data as well as self-report data. Here, we describe a prospective longitudinal study documenting quantitative and qualitative outcomes of a multi-departmental mentorship program at a single institution.

## Materials and methods

### Inclusion criteria

In 2009, a survey to faculty revealed interest in exploring formal mentorship, and with support of senior leadership, a pilot formal mentorship program was established at Massachusetts General Hospital (MGH) via the Center for Faculty Development (CFD). The CFD worked with an external consultant and representatives from the Departments of Radiation Oncology and Anesthesia, Critical Care and Pain Management to tailor a formal mentorship program. Junior and senior faculty members in the two departments were invited to participate. Mentor pairs were established by asking junior faculty mentees to rank the five areas of professional development most important to them and by asking senior faculty mentors to rank the five areas that they were best able to provide mentorship in, and then matching the most compatible faculty. Senior faculty members familiar with the mentees and mentors assisted with directing the pairings and securing mentor involvement. The control group for this study was comprised of junior faculty not in the Departments of Radiation Oncology or Anesthesia, Critical Care, and Pain Management and who completed the first questionnaire that was sent to all junior faculty at MGH. Institutional review board approval by the Partners Human Research Committee was obtained for this study and for obtaining informed consent and authorization from participants by voluntary completion of questionnaires.

### Program structure

The pilot formal mentorship program consisted of assignment of mentor pairs, three formal training sessions over the course of nine months, and regular informal meetings throughout the program. At the first two-hour training session, mentor pairs received an introduction to mentoring and formalized their action plan. Specifically, MGH leadership introduced the mission of the program, participants discussed best practices in a mentoring relationship, and mentor pairs created an action plan for their relationship that detailed joint expectations, guidelines and boundaries, strategies for addressing stumbling blocks, and goals. At the second two-hour training session, halfway through the pilot formal mentorship program, mentor pairs were given the opportunity to recalibrate their relationship and revisit their action plan. Participants discussed additional concepts of mentoring, such as emotional intelligence and phases of mentoring, worked through case scenarios of challenging mentoring situations, and mentor pairs discussed their relationships and revisited their commitments and goals. The final training session was a closing point for the formal mentoring relationships and served as an opportunity to set the stage for the transition to informal mentoring relationships. Participants discussed how to redefine their mentoring relationship, shared with other participants their best practices and plans for the future, and mentor pairs reflected on their mentoring relationship, on what was accomplished, and how they might work together moving forward. Mentor pairs were encouraged to have frequent informal meetings throughout the duration of the formal mentorship program as well as after its conclusion.

### Data collection

Faculty completed three questionnaires over the course of this study. The first questionnaire (S1 and S2 Files) was completed before beginning the formal mentorship program in 2009, the second (S3 and S4 Files) was completed immediately after the formal mentorship program ended, and the third (S5 File) was completed several years later in 2016. Results from the first and second questionnaire contributed to short-term assessment of the program, and results from the third questionnaire contributed to the long-term assessment of the program. Participants were emailed an individual link to the questionnaires that was administered using Survey Monkey.

The first and second questionnaires were divided into seven blocks of questions related to a specific aspect of mentoring. These blocks were “desire to be mentored,” “satisfaction with mentoring,” “personal achievement,” “satisfaction with work environment,” “challenges to mentoring,” “importance of attributes of mentor,” and “department fairness and cohesiveness.” The scale of responses to the questionnaire was a 5-point ordinal scale with two additional categories of “not applicable” and “too early to tell.” Depending on the nature of a particular question, the ordinal scale ranged from 1, indicating “not important, not satisfied or strongly disagree” to 5, indicating “extremely important, extremely satisfied or strongly agree.” The questionnaires were constructed by faculty specialized in faculty development by closely adapting common questionnaire format and content to generate questionnaires specific to our novel intervention, and thus were not previously validated questionnaires. The third questionnaire was administered to the faculty to assess subjective long-term impact of the pilot program and their current experiences as a mentee and a mentor, if applicable, in eight domains applicable to mentoring. Depending on the nature of each question, respondents either described the impact of the program on each domain by choosing a response from a 5-point ordinal scale and an additional category “not applicable,” or chose any domains that applied to a particular question. Respondents were also allowed to provide open-ended responses to describe why they did not continue an old mentoring relationship or establish a new mentoring relationship.

Long-term assessment of the effects of the mentorship program was comprised of subjective results from the third questionnaire, as well as objective data collected seven years after the beginning of the formal mentorship program. Retention rate data, former and current academic rank of participants, and grant funding information of the mentees participating in the mentoring program and the control junior faculty who remained at MGH were collected.

### Analysis

The block of questions in the first and second questionnaire contained between 7 and 21 individual sub-categories, and the sum of responses for each response score was tabulated to give an overall weight of that response score in the block. Response scores were also analyzed by placing them into two groups, with the “unsatisfactory” group containing response scores 1–3 and the “satisfactory” group containing response scores 4 and 5. The division of response scores into two groups better enabled tracking changes in responses between the two groups from before the mentoring program to after the program as well as between the study and control cohorts. The satisfactory and unsatisfactory groups were converted to proportions, and a Wald 95% confidence interval was generated for each proportion. To determine whether the median response within each group of questions was the same at baseline as after the program, we used a nonparametric Wilcoxon-Mann-Whitney U test. We used the Kolmogorov-Smirnov two-sample test to check the validity of the assumption that the distributions of responses at baseline and after the program were the same. Exact p values were calculated for both tests. In addition, for each group of questions, we tested the hypothesis that the proportion of satisfactory group responses was higher after the program than it was at baseline using a one-sided exact test for binomial random variable to test improved responses. All other tests were two sided. P < 0.05 was deemed statistically significant for all tests. Odds ratios (OR) and 95% confidence intervals were calculated to determine the likelihood of an improved score in the post-mentoring group.

The response scores from the third questionnaire were also placed into unsatisfactory and satisfactory groups. Because of the small sample size, only a descriptive summary of the results from the subjective third questionnaire is provided. The mentee and control cohort were compared using the Fisher’s exact test for categorical variables and the Wilcoxon rank-sum test for continuous variables. The independence of categorical variables was evaluated with the Pearson’s chi-squared test. Mentees were defined as holding junior ranking if they were a Lecturer, Instructor, or Assistant Professor, and senior ranking if they were an Associate Professor or Full Professor. To holistically assess any impact the mentoring program may have had on overall success in academic medicine, the metric “funded + promoted” was devised to sum the number of times a faculty member was promoted since the pilot mentorship program with either 1 or 0 if the faculty member had or had not received any funding, respectively.

## Results

### Short-term assessment

The 23 mentees that participated in the pilot mentoring program were contacted and administered the first survey before the start of the mentoring program. Three mentees subsequently left the mentoring program, and of the 20 remaining mentees, 14 (70%) responded to the second questionnaire sent at the end of the mentoring program. Of the 91 junior faculty members not in the mentoring program that completed the first questionnaire that was administered before the start of the mentoring program, a random sample of 50 was sent the second questionnaire after the end of the mentoring program, and 40 responded (80%).

**Fig 1:**
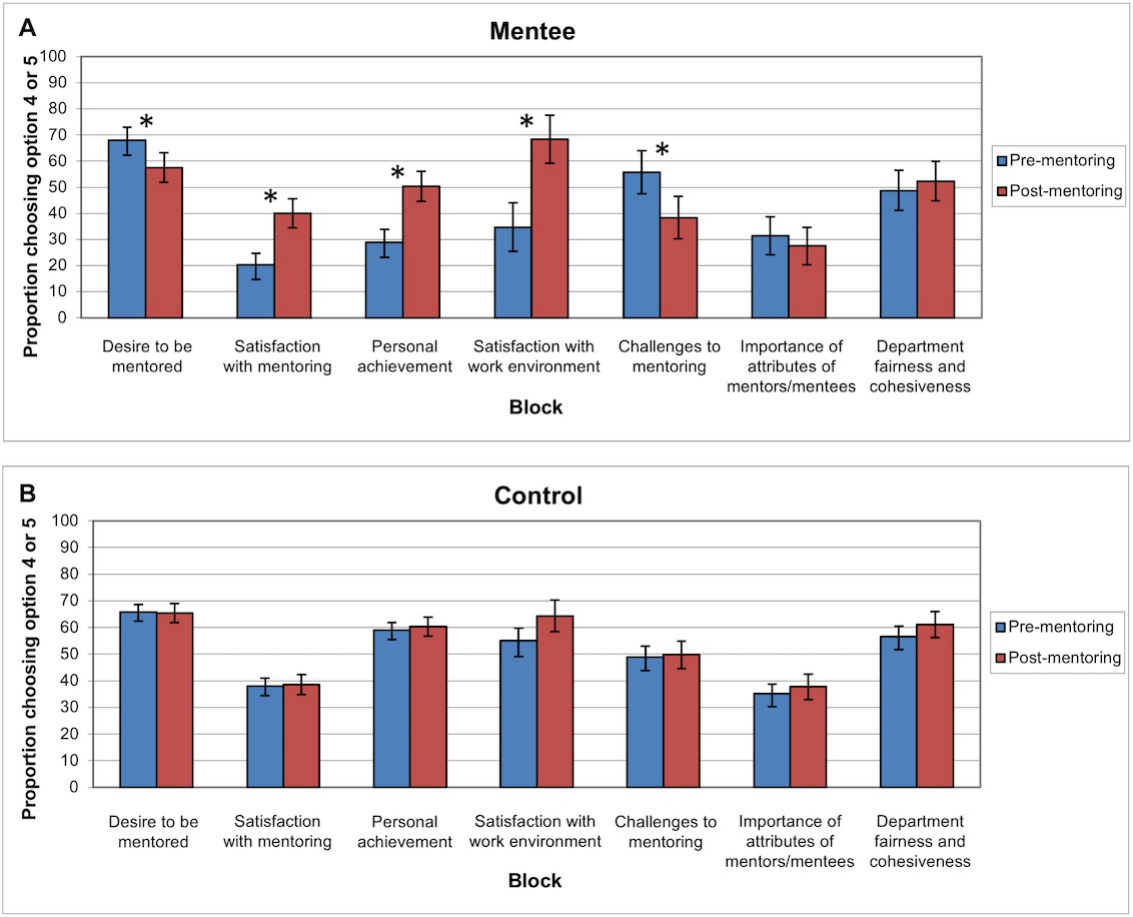
Perspectives of mentoring before and after faculty mentoring pilot program. (A) Mentees and (B) control faculty perspectives of several aspects of mentoring were assessed before and after the faculty mentoring pilot program. Twenty-three mentees completed the pre-mentoring questionnaire, and 14 of 20 remaining mentees completed the post-mentoring questionnaire. Ninety-one control faculty completed the pre-mentoring questionnaire, and 40 of 50 sampled control faculty completed the post-mentoring questionnaire. Depending on the nature of the question, option 4 indicated “important,” “satisfied,” or “agree,” and option 5 indicated “extremely important,” “extremely satisfied,” or “strongly agree”.

Within the mentee group, satisfaction after the mentoring program in blocks 1–5 was significantly improved from baseline (Fig 1). Ten percent fewer mentees reported a strong desire to be mentored following participation with the program (OR 0.6, 95% CI (0.46–0.89), p = 0.003). The proportion of responses indicating satisfaction with their mentoring experience increased from 20% to 40% (OR 2.6, (1.82–3.75), p<0.001), and satisfaction with the personal achievement that the mentee had achieved in their career rose from 29% to 50% (OR 2.5, (1.79–3.50), p<0.001). The largest increase in satisfactory responses, from 35% to 65% (OR 4.1, (2.24–7.38), p<0.001), reflected the mentees’ improved view of their workplace as an environment that promotes a culture of mentorship through increased feedback, visibility, guidance, and access to resources. Lastly, perceived challenges to a mentoring relationship, such as generational differences or lack of time, were reduced from 56% to 36% (OR 0.5, (0.31–0.80), p = 0.002). Responses in blocks six and seven, “the importance of attributes of mentors/mentees” and “the openness and fairness of the mentee’s department,” did not significantly change, from 31% and 49% pre-mentoring to 38% and 52% post-mentoring, respectively. The control group experienced no significant change from baseline to after the mentoring program in six of the seven domains, and 9% of controls reported an increase in satisfaction with their work environment (OR 1.5, (1.07–2.03), p = 0.009).

At baseline, we found that the distribution of responses and the median responses of the mentee and control groups were not statistically different except for in blocks 2 and 3, the domains describing satisfaction with mentoring and personal achievement. In these blocks, mentees demonstrated a lower satisfaction with mentoring (p = 0.01) and personal achievement (p = 0.001) than the control group. One year after the start of the mentoring program, there was no statistically significant difference between the mentee group and the control group across all question blocks. For blocks 2 and 3, the mentee group started at baseline with a lower proportion of “satisfactory” answers than the control group, but ended up with a similar proportion of “satisfactory” answers compared to the control group after the program was completed.

### Long-term assessment

**Fig 2:**
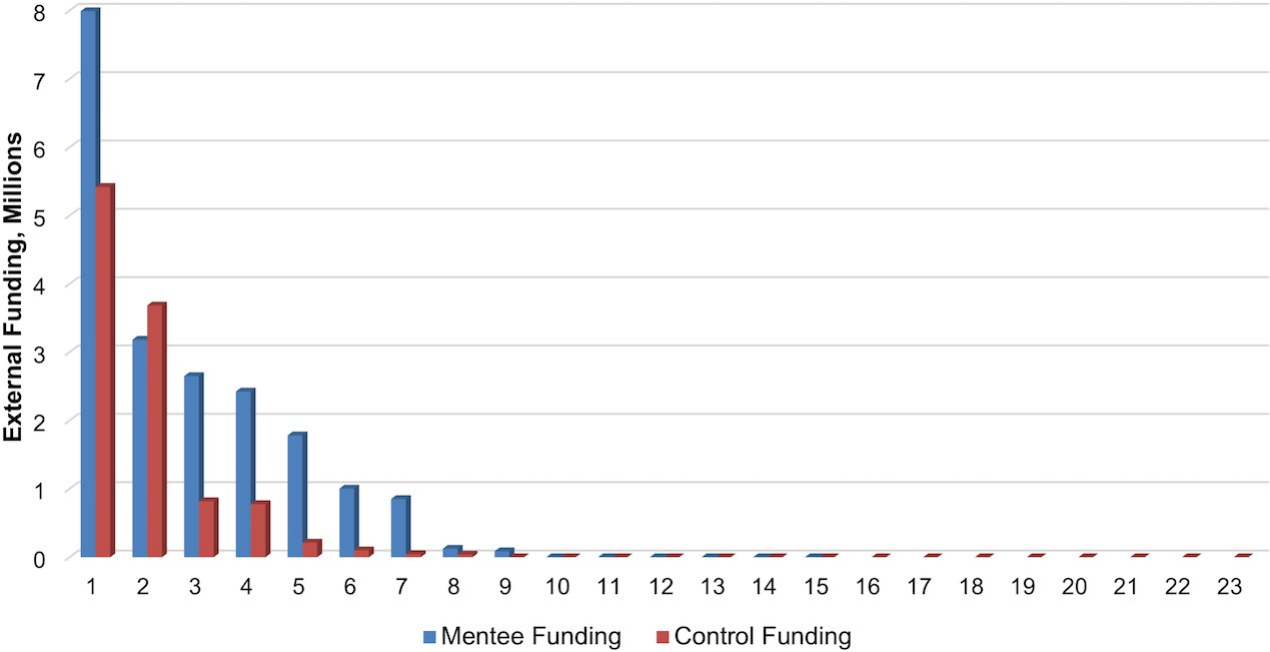
External funding. For all 15 mentees and 23 control faculty that remained at MGH at long-term follow up, all individual external funding received from baseline to long-term follow up is displayed, in millions of dollars.

Of the 40 responding control faculty, only 32 provided identification, which was necessary for long-term follow up. The institution retention rate in 2016 for mentees was 15/20 (75%) and for controls was 23/32 (72%). Only the faculty that remained at MGH were assessed for long-term academic promotion and grant funding amounts. Mentees were more likely to hold senior faculty positions at long-term follow up (p = 0.030) despite no difference in initial rank (p = 0.22) or other baseline characteristics (Table 1). Specifically, 7 out of 15 (47%) mentees were Associate Professors at long-term follow up, whereas 3 out of 23 (13%) controls were Associate Professors. To assess whether this difference could be explained by differences in specific initial academic rank, a chi-squared test of independence was performed to examine the relation between Assistant Professor ranking at baseline with group assignment, but no relationship was found (X^2^ (1, N = 38) = 2.2488, p = 0.134). Mentees received a median external funding amount of $1,783,200 and controls received a median of $495,900 in external funding, but this difference did not reach statistical significance (p = 0.21). Fig 2 displays all individual external funding amounts for mentees and controls. However, when comparing faculty who were the same academic rank of Instructor at baseline, there was a significant relationship between group status and the metric “funded + promoted”, X^2^ (3, N = 29) = 8.9514, p = 0.030, with mentees being more likely to be funded and/or promoted than controls. When examined separately, neither promotion nor funding reached a significant association with group status.

**Table 1:** Demographic characteristics.

	Control	Mentee	*p*
Number of faculty	23	15	-
Sex, n (%)			0.32
Female	14 (61%)	6 (40%)	
Male	9 (39%)	9 (60%)	
Initial Rank, n (%)			0.22
Lecturer	1 (4%)	0 (0%)	
Instructor	19 (83%)	10 (67%)	
Assistant Professor	3 (13%)	5 (33%)	
Final Rank, n (%)			0.12
Lecturer	1 (4%)	0 (0%)	
Instructor	13 (57%)	6 (40%)	
Assistant Professor	6 (26%)	2 (13%)	
Associate Professor	3 (13%)	7 (47%)	
Final Junior or Senior Rank, n (%)			**0.030**
Junior Faculty	20 (87%)	8 (53%)	
Senior Faculty	3 (13%)	7 (47%)	
Promoted, n (%)			0.32
No	15 (65%)	7 (47%)	
Yes	8 (35%)	8 (53%)	
Times Promoted, n (%)			0.35
0	15 (65%)	7 (47%)	
1	7 (30%)	5 (33%)	
2	1 (4%)	3 (20%)	
Funding, n (%)			0.19
No	15 (65%)	6 (40%)	
Yes	8 (35%)	9 (60%)	
Funding Amount, ($K)			0.21
median (IQR)	495.9 (72.5, 2250.9)	1783.2 (851.8, 2649.3)	
Funded + Promoted			0.18
0	12 (52%)	5 (33%)	
1	5 (22%)	3 (20%)	
2	6 (26%)	4 (27%)	
3	0 (0%)	3 (20%)	

**Fig 3:**
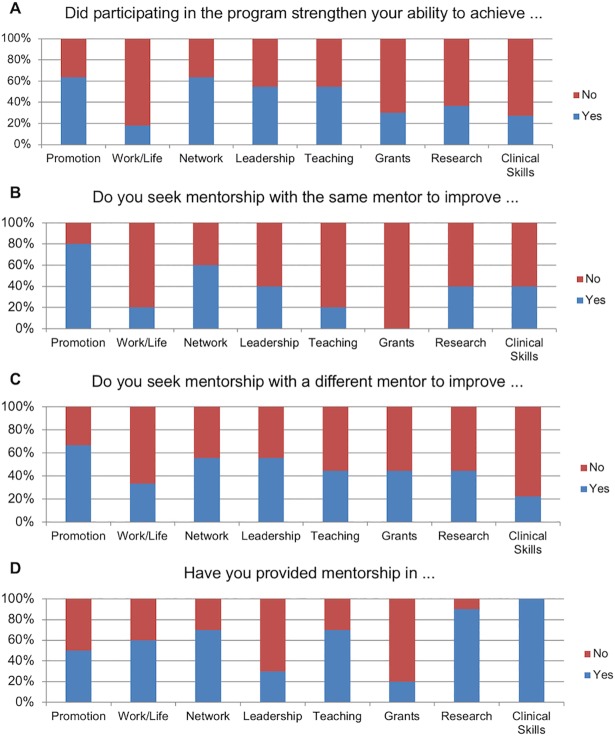
Subjective perspectives on long term impact of mentoring program. Mentees that remained at MGH at long term follow up were assessed for their subjective perspectives on the long-term impact of the faculty mentoring pilot program on (A) their ability to succeed in several domains (n = 10), (B) their relationship with their assigned mentor (n = 5), (C) their relationship with other mentors (n = 9), and (D) their experiences as a mentor (n = 10).

Of the 15 mentees remaining at MGH, 11 (73%) responded to the third long-term subjective questionnaire in 2016. A majority of respondents stated that they thought that the pilot mentorship program in 2009 had strengthened their ability to achieve academic promotion (7/11), access a larger professional network and obtain increased professional visibility (7/11), obtain more leadership positions (6/11), and serve as a teacher and mentor (6/11) (Fig 3). Five mentees reported that they still receive mentorship from the original mentor that they met with during the pilot program, and primarily sought guidance in achieving academic promotion (4/5) and increasing their professional network (3/5). The other six mentees who did not continue their original mentoring relationships commonly cited lack of time and access to other mentors as contributing reasons. Specifically, nine mentees reported forming mentoring relationships with new mentors, and they also sought mentorship in achieving academic promotion (6/9) and broadening their professional network (5/9), as well as in obtaining leadership positions (5/9). Since the pilot mentorship program, ten of the eleven mentees became mentors themselves of a combined 57 mentees. The majority of the original mentees provided mentorship in numerous areas, including in achieving promotion (5/10), gaining a broader professional network (7/10), enabling a work/life balance (6/10), in serving as a teacher and mentor (7/10), in publishing more research (9/10), and developing clinical skills (10/10).

## Discussion

Recent reports have demonstrated tangible benefits of a mentorship program on faculty development. Our study builds on these recent reports by examining several domains of mentorship. Unlike many previous reports, our study consisted of a prospective long-term comparison of mentees and a control population in both subjective and objective areas, and allows for novel observations. We found that faculty who participated in the formal mentorship program derived both short-term and long-term benefits in comparison to faculty who did not. Mentees reported subjective improvements in their productivity and satisfaction in many areas, and demonstrated higher objective achievement than controls.

To assess the validity of comparing the faculty who participated in the pilot mentoring program with the control faculty, we evaluated baseline perceptions of mentoring and academic rank for both groups. We found that there was no statistical difference in desire to be mentored, satisfaction with their work environment, perceptions of challenges in being mentored, ascribed importance of mentor attributes, nor perception of department fairness and cohesiveness. Finally, there was no statistical difference in baseline academic rank of mentees compared to control faculty. Thus, given the several baseline similarities of the mentee and control faculty with regards to their perceptions of their department, their expectations of mentoring, and their objective achievement, we found it appropriate to compare the short-term and long-term differences between the two groups.

We found that upon concluding the pilot mentorship program, mentee attitudes changed significantly from baseline. Among other changes, mentee satisfaction with level of personal achievement increased from 29% to 50%, and their satisfaction with their work environment increased from 35% to 65%. While these are significant improvements, there is still room for improvement in the overall level of satisfaction in each domain. The control group, on the other hand, experienced no significant changes in any domains from the beginning of the mentoring program to the end other than a 9% increase in satisfaction with their work environment. The positive externalities of the pilot mentorship program appear to persist for several years, and these same domains remain a focus for the mentees. A majority of mentees still work with mentors to increase their ability to achieve promotion, leadership positions, and a broader professional network, and most mentees cite participation in the pilot mentorship program as integral to their improvement in these domains. The subjective intuition of the mentees is supported by long-term follow up data; despite no difference in baseline academic rank, by 2016, mentees were more likely to hold senior rankings than controls. Mentees received over triple the amount of median external funding than controls, and while this difference did not meet statistical significance, it trends towards importance. By adjusting for initial ranking by only comparing faculty who were Instructors at baseline, mentees were found to be more likely to be funded and/or promoted than controls. While separately there was no difference between groups in receiving a promotion or in receiving funding, pooling these two important indicators of success in this relatively small cohort may allow for the detection of the holistic effects of a mentoring program on overall success in academic medicine. Several years after the conclusion of the pilot mentoring program, five of 11 responding mentees continued their original mentoring relationship, and nine secured additional mentors to assist them with their evolving goals. Importantly, the vast majority of mentees became mentors themselves to a combined 57 mentees, highlighting the strong culture of mentorship that was instilled.

Mentorship is widely regarded as important to the fostering of young faculty at centers of academic medicine. However, these institutions and the senior faculty who would serve as mentors have limited time and funding, and thus mentoring may be underutilized without clear evidence of its benefits [13,14]. Here, we suggest several tangible benefits resulting from a structured program that addresses concerns raised in previous studies. It is still unclear whether more benefit is derived when mentees are paired with mentors, or when mentees can choose their own mentors [2]. The program established at our institution sought to facilitate the likelihood of a compatible pairing by matching mentees and mentors based on their responses to holistic surveys about how the mentoring relationship would best suit their own goals and expertise. Another benefit of a formal mentorship program is that it provides guidance to mentors on how to be an effective mentor, including how to develop joint expectations, goals, and an appropriate timeline. A hallmark of quality mentoring relationships is their durability, and roughly half of the mentoring relationships persisted for many years [1,15]. The relationships that faded in our study did so because over the course of several years, per self-report, mentees developed goals that outgrew their mentor’s expertise. Despite the prevalence of reports on mentoring at centers of academic medicine, conclusions are often limited by the ambiguity of the structure and benefits of those mentoring programs [2,4]. In contrast, the intervention implemented by this pilot program was straightforward and reproducible, and its qualitative and quantitative effects on the prospectively enrolled subject and control populations were carefully documented at varying time points. Specifically, we have provided details on the structure of the formal mentorship program, the content and agenda of all training sessions, the questionnaires used in this study, and the benefits that were derived from this intervention. This specificity and clarity should allow for the described intervention to be readily reproduced at other institutions that want to establish a formal mentoring program and realize similar benefits to faculty development.

Our study has some limitations. Mentees came from only two departments, the Department of Radiation Oncology and the Department of Anesthesia, Critical Care and Pain Management. Holliday et al. examined mentorship in radiation oncology, and highlighted that the improvements seen in their study may be attributed to the fact that the competitiveness of radiation oncology necessitates that faculty utilize any available resource, such as mentoring, to not fall behind their peers [11]. Furthermore, there can be inherent differences in the personality or lifestyle of physicians in different fields, and thus in their ability to be mentored. In other words, the benefits of mentoring programs may vary based on the targeted audience and the relative advantages that a mentoring relationship may provide. Our study could also have been limited by unmeasured confounders such as faculty development efforts that were independent from the formal mentorship program. Another limitation of this study is the possibility of response bias by the control group faculty who responded to our surveys, although there were no differences in several baseline characteristics between the control group and the mentee group. Our study examined a relatively small cohort of subjects from a single institution, which may limit the ability to generalize our findings, although we did have good response rates from this cohort. This study would be more powerful if we examined mentees from more diverse departments and at multiple institutions, although the mentoring program was a pilot program that intentionally addressed a smaller scope.

In summary, we report several benefits derived from a reproducible intervention that was aimed at improving the quality of mentoring relationships. This study provides evidence to support the adoption and expansion of mentoring programs at other institutions to help propagate a culture of mentorship and excellence.

## Supporting information

S1 File Mentee pre-program mentorship questionnaire. The first questionnaire sent to mentees before the formal mentorship program began.
(PDF)

S2 File Control pre-program mentorship questionnaire. The first questionnaire sent to controls before the formal mentorship program began.
(PDF)

S3 File Mentee post-program mentorship questionnaire. The second questionnaire sent to mentees after the formal mentorship program ended.
(PDF)

S4 File Control post-program mentorship questionnaire. The second questionnaire sent to controls after the formal mentorship program ended.
(PDF)

S5 File Long-term mentorship questionnaire. The third questionnaire sent to mentees several years after the formal mentorship program ended.
(PDF)
