# Supplementary material for: Long-term impact of a faculty mentoring program in academic medicine
Source: PLoS One. 2018 Nov 29;13(11):e0207634. doi: 10.1371/journal.pone.0207634 (PMC6264475; doi:10.1371/journal.pone.0207634)
Supplement: S1 File — The first questionnaire sent to mentees before the formal mentorship program began. (PDF) [file pone.0207634.s001.pdf]

Mass General Mentorship Pilot Program Survey--Mentee

1.

* 1. Please rank the following according to your present commitment

	1-most, 5-least
Admin/Committee Work	
Patient Care	
Teaching	
Research	
Other	

2.

Please note the scale options with each question

* 1. Prior to the Monday January 12th mentorship pilot program session, did you already have someone at MGH you considered to be a mentor?

- Yes
- No

2. If yes to the question above, how satisfied were you in being mentored?

- too early to tell
- not satisfied
- mildly satisfied
- moderately satisfied
- very satisfied
- extremely satisfied
- not applicable

* 3. Do you personally have a role model?

- Yes
- No

3.

Please note the scale and question in each of the options below.

Mass General Mentorship Pilot Program Survey--Mentee

* 1. Prior to the Monday January 12th mentorship pilot program session, how important was it to you to be mentored in the following areas?

Please note the options include a scale of "not important" to "extremely important." In addition, included are options "too early to tell" and "not applicable"

	too early to tell	not important	mildly important	moderately important	very important	extremely important	not applicable
Ability to give feedback							
Ability to receive feedback							
Academic promotion							
Balancing work and family Life							
Career planning and advice							
Career success							
Clinical skills							
Communication skills							
Increasing your visibility							
Integrating research and clinical activities							
Job satisfaction							
Knowledge about the HMS and MGH organizational system and culture							
Leadership skills							
Learning how to be a mentor/mentoring skills							
Networking							
Opportunities for self reflection							
Professional connectedness							
Scientific research/grant writing							
Self confidence							
Teaching							
Time management							

Mass General Mentorship Pilot Program Survey--Mentee

* 2. Prior to the Monday January 12th mentorship pilot program session, how satisfied were you that you personally had been mentored in the following areas?

Please note the options include a scale of "not satisfied" to "extremely satisfied." In addition, included are options "too early to tell" and "not applicable"

	too early to tell	not satisfied	mildly satisfied	moderately satisfied	very satisfied	extremely satisfied	not applicable
Ability to give feedback							
Ability to receive feedback							
Academic promotion							
Balancing work and family life							
Career planning and advice							
Career success							
Clinical skills							
Communication skills							
Increasing your visibility							
Integrating research and clinical activities							
Job satisfaction							
Knowledge about the HMS and MGH organizational system and culture							
Leadership skills							
Learning how to be a mentor/mentoring skills							
Networking							
Opportunities for self reflection							
Professional connectedness							
Scientific research/grant writing							
Self confidence							
Teaching							
Time management							

Mass General Mentorship Pilot Program Survey--Mentee

* 3. Prior to the Monday January 12th mentorship pilot program session, to what extent do you agree you personally have achieved the following in your career?

	too early to tell	strongly disagree	disagree	neither agree nor disagree	agree	strongly agree	not applicable
Ability to give feedback							
Ability to receive feedback							
Academic promotion							
Balancing work and family life							
Career planning and advice							
Career success							
Clinical skills							
Communication skills							
Increased visibility							
Integrating research and clinical activities							
Job satisfaction							
Knowledge about the HMS and MGH organizational system and culture							
Leadership skills							
Learning how to be a mentor/mentoring skills							
Networking							
Opportunities for self reflection							
Professional connectedness							
Scientific research/grant writing							
Self confidence							
Teaching							
Time management							

4.

Mass General Mentorship Pilot Program Survey--Mentee

1. Prior to the Monday January 12th mentorship pilot program session, to what extent do you agree the following were present at work?

Please note the options include a scale of "strongly disagree" to "strongly agree." In addition, included are options "too early to tell" and "not applicable"

	too early to tell	strongly disagree	disagree	neither agree nor disagree	agree	strongly agree	not applicable
Environment that promotes a culture of mentorship							
Environment that promotes feedback and sharing of information							
Environment that promotes visibility							
Environment that provides actionable advice and guidance							
Knowledge of available departmental information							
Knowledge of how to access departmental resources							
Someone to turn to in case of difficulty							

5.

Mass General Mentorship Pilot Program Survey--Mentee

* 1. Prior to the Monday January 12th mentorship pilot program session, to what extent do you agree the following are challenges to a mentoring relationship?

Please note the options include a scale of "strongly disagree" to "strongly agree." In addition, included at are options "too early to tell" and "not applicable"

	too early to tell	strongly disagree	disagree	neither agree nor disagree	agree	strongly agree	not applicable
Different goals							
Generational differences							
Giving honest feedback							
Lack of perceived confidentiality							
Mentor's lack of time							
Perception that your mentor might have a role in evaluating your job performance							
Personality conflict							
Receiving honest feedback							
Mentor's lack of experience							
Your lack of time							

6.

Mass General Mentorship Pilot Program Survey--Mentee

* 1. Prior to the Monday January 12th mentorship pilot program, if you were to make suggestions in designing a mentorship program, how important are the following in matching mentees with mentors?

	too early to tell	not important	mildly important	moderately important	very important	extremely important	not applicable
Ability to choose one's own mentor							
Ability to choose one's own mentee							
Administrative interests							
Age							
Alignment of goals							
Gender							
Marital status							
Parental status							
Personality							
Research interests							
Specialty/Subspecialty							

7.

* 1. Prior to the Monday January 12th mentorship pilot program session, please rate the extent to which you agreed with the following statement:

by encouraging mentorship, the Mass General and my department show they value my own professional development

- too early to tell
- strongly disagree
- disagree
- neither agree nor disagree
- agree
- strongly agree
- not applicable

8.

Mass General Mentorship Pilot Program Survey--Mentee

* 1. Prior to the Monday January 12th mentorship pilot program session, to what extent did you agree that you were supported, in general?

- too early to tell
- strongly disagree
- disagree
- neither agree nor disagree
- agree
- strongly agree
- not applicable

9.

Mass General Mentorship Pilot Program Survey--Mentee

* 1. Prior to the Monday January 12th mentorship pilot program session, please rank the extent to which you agree the following statements describe your department at this time:

	too early to tell	strongly disagree	disagree	neither agree nor disagree	agree	strongly agree	not applicable
Allocation of rewards by your department is linked to measures of individual faculty performance.							
Faculty in your department are committed to the decisions made in the department.							
Faculty in your department are not distracted by continual shifts in policy or objectives.							
Faculty in your department feel they are part of a close-knit team.							
Faculty in your department share information.							
Faculty in your department strive to support each other.							
Faculty who are not academically and/or clinically productive are not successful in your department.							
In your department, individual faculty performance is monitored against written objectives.							
In your department, there is an emphasis on innovative/new/cutting edge activities.							
In your department, there is an emphasis on measurement of individual faculty performance.							
In your department, your work is consistent with your expectations.							
Your department is open to change.							
