# Supplementary material for: Long-term impact of a faculty mentoring program in academic medicine
Source: PLoS One. 2018 Nov 29;13(11):e0207634. doi: 10.1371/journal.pone.0207634 (PMC6264475; doi:10.1371/journal.pone.0207634)
Supplement: S5 File — The third questionnaire sent to mentees several years after the formal mentorship program ended. (PDF) [file pone.0207634.s005.pdf]

Long Term Follow-up to MGH Faculty Mentorship Program

Thank you for your time to complete this approximate three-minute survey.

Once again, please note that you can only access this short survey once, so please be prepared to answer all four questions.

Long Term Follow-up to MGH Faculty Mentorship Program

* 1. Do you feel as though participating in the pilot mentorship program has strengthened your ability to....
(Please mark the appropriate section)

	Strongly Agree	Agree	Neither Agree or Disagree	Disagree	Strongly Disagree	N/A
...Achieve academic promotion?	○	○	○	○	○	○
...Achieve work/ life balance?	○	○	○	○	○	○
...Access a larger professional network and obtain increased professional visibility?	○	○	○	○	○	○
...Achieve more leadership positions?	○	○	○	○	○	○
...Serve as a teacher and mentor?	○	○	○	○	○	○
...Obtain higher levels of independent grant funding?	○	○	○	○	○	○
...Publish more research?	○	○	○	○	○	○
...Develop clinical skills?	○	○	○	○	○	○

Long Term Follow-up to MGH Faculty Mentorship Program

* 2. Do you still seek mentorship/guidance from the mentor you met with during the program?

○ Yes

○ No

In what specific areas have you sought mentorship? *(Please mark all that apply)*

- [ ] Achieving academic promotion
- [ ] Achieving work/life balance
- [ ] Accessing a larger professional network and obtaining increased professional visibility
- [ ] Achieving more leadership positions
- [ ] Serving as a teacher and mentor
- [ ] Obtaining higher levels of independent grant funding
- [ ] Publishing more research
- [ ] Developing clinical skills
- [ ] Other (please specify)

Long Term Follow-up to MGH Faculty Mentorship Program

Why do you not seek mentorship/guidance from your previous mentor?

Long Term Follow-up to MGH Faculty Mentorship Program

* 3. Since the pilot mentorship program, have you reached out and established relationships with other mentors?

- ○ Yes
- ○ No

Long Term Follow-up to MGH Faculty Mentorship Program

In what specific areas have you sought mentorship? *(Please mark all that apply)*

- [ ] Achieving academic promotion
- [ ] Achieving work/life balance
- [ ] Accessing a larger professional network and obtaining increased professional visibility
- [ ] Achieving more leadership positions
- [ ] Serving as a teacher and mentor
- [ ] Obtaining higher levels of independent grant funding
- [ ] Publishing more research
- [ ] Developing clinical skills
- [ ] Other (please specify)

Long Term Follow-up to MGH Faculty Mentorship Program

Why have you not reached out to other potential mentors?

Long Term Follow-up to MGH Faculty Mentorship Program

* 4. Since the pilot mentorship program, have you acquired your own mentees to whom you provide mentorship?

- ( ) Yes
- ( ) No

Long Term Follow-up to MGH Faculty Mentorship Program

* How many mentees?

Long Term Follow-up to MGH Faculty Mentorship Program

In what specific areas do you provide mentorship? *(Please mark all that apply)*

- ☐ Achieving academic promotion
- ☐ Achieving work/life balance
- ☐ Accessing a larger professional network and obtaining increased professional visibility
- ☐ Achieving more leadership positions
- ☐ Serving as a teacher and mentor
- ☐ Obtaining higher levels of independent grant funding
- ☐ Publishing more research
- ☐ Developing clinical skills
- ☐ Other (please specify)

Long Term Follow-up to MGH Faculty Mentorship Program

Why have you not acquired your own mentees?

Long Term Follow-up to MGH Faculty Mentorship Program

Thank you for taking the time to complete this survey. We wish you the best in your personal and professional journey.
